# The Exposition

**Published:** 1901-06

**Authors:** 


A Monthly Review of Medicine and Surgery.

*EDITOR:*
WILLIAM WARREN POTTER, M. D.
All communications, whether of a literary or business nature, books for review and exchanges should be addressed to the editor: 284 FRANKLIN STREET, BUFFALO, N. Y.

VOL. XL.—LVI. JUNE, 1901. NO. 11.

### THE EXPOSITION.

THE Pan-American Exposition was opened in Buffalo, according to schedule, May 1, and was duly dedicated, with all the "pomp and circumstance" due the great occasion, on the 20th day of May, 1901. That such a gigantic undertaking has been planned, constructed, and paid for within a little more than two years, does credit to the men who conceived it, nurtured it, and finally brought it forth in all its Andalusian splendor. Moreover, it is a credit to the good city of Buffalo, that its citizens should have done all this and paid for it out of their own pockets; for it is a well-known fact that there is not a dollar owing as a result of all this outlay, except to the people themselves, who so generously contributed to the capital stock of the company.

It is even a source of comfort, although, perforce, one of disappointment at the time, that the appropriation from congress failed in the closing hours of the session. It has made the exposition self-reliant and independent; whatever it is, it is indebted to no subsidiary aid, but owns itself, so to speak.

Probably no exposition was ever ideally complete at its opening or dedication; probably, likewise, none was ever more so than the Pan-American Exposition of 1901. Whether one looks at it from an engineering, architectural, artistic, business, or pleasure viewpoint, there is little to criticise, but much to praise. In some of its features it is even unique.

One of the most striking points to the stranger is the marvelous color effect that pervades the whole field of vision. Never before has this been applied so effectually or so effectively to buildings of this character. It is this that has so appropriately named the exposition group of buildings, The Rainbow City. Indeed, the coloring is so well done and so exquisitely blended that it even puts the rainbow out of commission as a competitor.

It has been said that in six important features this will surpass all previous expositions ever held: first, in court settings; second, in hydraulic and fountain effects of wonderful beauty and extent; third, in horticultural and floral embellishments; fourth, in color decorations; fifth, in plastic and sculptural decorations; sixth, in electrical effects. Whether this be absolutely true in all respects we know not, but we are quite certain that in all these points no visitor will be disappointed in his expectations. Moreover, in all of them a superior excellence will be found; while in two, color and electrical effects, it is far and away the best that has ever been produced.

As an educator the exposition is in its strongest pose and presents its mightiest appeal to the people, young or old. It is there that art, engineering, landscape and building architecture, machinery, botany, electricity, mechanics and numerous other topics, can be studied at their best advantage, and in this way benefit can be derived of a lasting nature by a visit to its realms. This point is capable of great enlargement had we adequate space at command.

Another essential feature of the exposition is its relation to political economics. It ought to, and no doubt will, prove a bond of stronger union between Mexico, Central and South America, our island possessions and ourselves. Through it may be established a reciprocity in commercial dealings, that ought to benefit all the countries named, and advance the influence of the western hemisphere in the great enterprises of the world. It ought, too, to divert the swarm of young people this way, who have heretofore sought Europe for educational purposes. Our colleges, schools, and shops furnish unexcelled opportunities for academic, professional or artisan training, and, as nearer neighbors to Latin America, we should establish educational relations with it at an early day.

Lastly, the exposition presents amusements for the millions who will visit it, of a most attractive and innocent variety. Its midway is more extensive than any exposition has heretofore presented, and it is free from any objectionable features. A visit to the exposition will pay.

